# Characterizing Droughts During the Rice Growth Period in Northeast China Based on Daily SPEI Under Climate Change

**DOI:** 10.3390/plants14010030

**Published:** 2024-12-25

**Authors:** Tangzhe Nie, Xiu Liu, Peng Chen, Lili Jiang, Zhongyi Sun, Shuai Yin, Tianyi Wang, Tiecheng Li, Chong Du

**Affiliations:** 1 School of Water Conservancy and Electric Power, Heilongjiang University, Harbin 150080, China; 2019036@hlju.edu.cn (T.N.); 2222019@s.hlju.edu.cn (X.L.); jianglili@hlju.edu.cn (L.J.); 2 College of Agricultural Science and Engineering, Hohai University, Nanjing 211100, China; 3 College of Ecology and Environment, Hainan University, Haikou 570208, China; 4 State Key Laboratory of Remote Sensing Science, Aerospace Information Research Institute, Chinese Academy of Sciences, Beijing 100101, China; 5 School of Water Conservancy and Civil Engineering, Northeast Agricultural University, Harbin 150030, China; 6 Institute of Geographic Sciences and Natural Resources Research, Chinese Academy of Sciences, Beijing 100101, China

**Keywords:** rice, potential evapotranspiration (*PET_0_*), standardized precipitation evapotranspiration index (*SPEI*), climate change, drought characteristics

## Abstract

In agricultural production, droughts occurring during the crucial growth periods of crops hinder crop development, while the daily-scale standardized precipitation evapotranspiration index (*SPEI*) can be applied to accurately identify the drought characteristics. In this study, we used the statistical downscaling method to obtain the daily precipitation (*P_r_*), maximum air temperature (*T_max_*) and minimum air temperature (*T_min_*) during the rice growing season in Heilongjiang Province from 2015 to 2100 under the SSP1-2.6, SSP2-4.5 and SSP5-8.5 in CMIP6, to study the spatial and temporal characteristics of drought during the rice growing season in cold region and the effect of climate change on drought characteristics. The potential evapotranspiration (*PET_0_*) was calculated using the regression correction method of the Hargreaves formula recommended by the FAO, and the daily *SPEI* was calculated to quantitatively identify the drought classification. The Pearson correlation coefficient was used to analyze the correlation between the meteorological factors (*P_r_*, *T_max_*, *T_min_*), *PET_0_* and *SPEI*. The results showed that: (1) Under 3 SSP scenarios, *P_r_* showed an increasing trend from the northwest to the southeast, *T_max_* showed an increasing trend from the northeast to the southwest, and higher *T_min_* was mainly distributed in the east and west regions. (2) *PET_0_* indicated an overall interannual rise in the three future SSP scenarios, with higher values mainly distributed in the central and western regions. The mean daily *PET_0_* values ranged from 4.8 to 6.0 mm/d. (3) Under SSP1-2.6, rice mainly experienced mild drought and moderate drought (−0.5 ≥ *SPEI* > −1.5). The predominant drought classifications experienced were mild, moderate, and severe drought under SSP2-4.5 and SSP8.5 (−0.5 ≥ *SPEI* > −2.0). (4) The tillering stage experienced the highest drought frequency and drought intensity, with the longest drought lasting 24 days. However, the heading flower stage had the lowest drought frequency and drought intensity. The drought barycenter was mainly in Tieli and Suihua. (5) The *PET_0_* was most affected by the *T_max_*, while the *SPEI* was most affected by the *P_r_*. This study offers a scientific and rational foundation for understanding the drought sensitivity of rice in Northeast China, as well as a rationale for the optimal scheduling of water resources in agriculture in the future.

## 1. Introduction

The Intergovernmental Panel on Climate Change’s Sixth Assessment Report (IPCC AR6) forecasts a hastened pace of global warming, indicating that the rise in global temperature is anticipated to attain or surpass 1.5 °C [1]. Moreover, precipitation will experience a reduction of around 2% for each 1 °C increment in temperature [2,3]. Under this scenario, further alterations in the global water cycle will result in more intense precipitation and more severe droughts [4]. Droughts are more likely to deteriorate as a result of ongoing climate change and increased human activity, which presents serious dangers to the environment, agriculture and human livelihoods [5]. Agriculture is crucial for socio-economic development. Severe agricultural drought may imperil food security and social stability [6].

In the context of global warming, the climate has been affecting the global water cycle by altering the patterns of precipitation, temperature, surface runoff, potential evapotranspiration (*PET_0_*) and increasing atmospheric water vapor content [7]. These alterations generally result in a further intensification of various climate extremes, such as droughts, floods, heat waves, cold waves, and extreme precipitation events, with respect to their duration, frequency, and intensity [8]. Among these alterations, precipitation, temperature, and evapotranspiration are the primary factors contributing to the occurrence of drought events. For instance, Wang et al. conducted an assessment of the global drought characteristics by employing multiple indicators from the CMIP6 model. Their findings revealed that drought was expected to intensify in the majority of regions across the globe in the future, with the alterations in *PET_0_* being among the key contributing factors [9]. Ren et al. and Zeng et al. demonstrated that an increase in *PET_0_* would increase the frequency, duration and severity of droughts [10,11]. Gond et al. assessed the changes in drought in Northern India and indicated that drought prediction was highly reliant on the prediction of precipitation and temperature [12]. Ren et al. showed that the difference between precipitation and *PET_0_* would increase since *PET_0_* was significantly affected by the temperature increase [10].

Drought is a type of meteorological disaster that is marked by its high occurrence frequency, long duration, and extensive impact range. Notably, it exerts a particularly serious impact on agricultural production [13]. Generally, droughts can be categorized into four distinct types specifically meteorological drought, agricultural drought, hydrological drought, and socio-economic drought [14]. A considerable number of drought indices have been devised on the basis of diverse drought analysis parameters. Examples of these indices include the Palmer Drought Severity Index (*PDSI*), the Standardized Precipitation Evapotranspiration Index (*SPEI*), the Standardized Precipitation Index (*SPI*), and the Socio-Economic Drought Index (*SEDI*) [15,16,17]. Regarding the application of these drought indices, the *PDSI*, which is founded on soil water balance, demands relatively high-quality data. Moreover, it lacks the adaptability to accommodate the inherent multi-scale characteristics of drought. The *SPEI* and *SPI* are important indicators for the assessment of regional droughts in a warming climate and can be used to identify meteorological drought characteristics with multi-scale features [18]. Nonetheless, the *SPI* solely identifies drought based on precipitation. In contrast, the *SPEI* represents an improvement over *SPI*. The principal advantage of *SPEI* compared to *SPI* lies in its ability to identify alterations in evapotranspiration and temperature during drought assessment [19,20]. The *SPEI* has been extensively employed in the fields of drought monitoring and assessment [21,22]. Ling et al. employed *SPEI* to investigate the drought characteristics of maize within the growing period in the Huang-Huai-Hai Plain spanning from 1960 to 2020 and indicated that droughts in the maize seeding period were the most frequent and lasted the longest events. The lowest frequency and shortest duration of drought occurred during the jointing-flowering period, and the barycenter of drought shifted eastward from 1970 to 2000 [1]. Qin et al. used *SPEI* to study the effect of drought on sugarcane yield, and the conclusion showed that the drought stages of sugarcane were the seeding and tillering stages most heavily impacted [23]. Currently, numerous scholars have opted to employ *SPEIs* on larger time scales to characterize drought. However, the monthly-scale *SPEIs* exhibited relatively lower sensitivity towards short-term droughts that take place during the crop growth period [24,25]. When the drought is sporadic and transiently severe, diagnosing the drought on a monthly scale becomes challenging. Consequently, in order to more effectively disclose the drought characteristics during the crop growth period and accurately identify the short-term droughts that occur throughout this period, drought indices on short-term time scales ought to be utilized for monitoring and analysis [1].

A noticeable trend of drought has happened in Northeast China in recent years, with a notable increase in the frequency and intensity of droughts. This has negatively impacted both national food security and agricultural productivity due to the uneven spatial and temporal distribution of precipitation and the ongoing rise in temperature [26]. Heilongjiang Province is a major grain-producing region in Northeastern China’s Songnen Plain [27]. By 2020, 289.615 million tons of rice were produced, corresponding to 13.67% of the country’s total [28]. Changes in precipitation and temperature have been found to affect rice yields in a number of studies [29,30]. A decrease in rice yields, for instance, has been attributed to warmer temperatures along with greater precipitation, suggesting that rice production is extremely susceptible to floods and drought events brought on by climate change [31]. Currently, the controlled irrigation mode is being promoted in Heilongjiang Province. Under this mode, except for the returning green stage, no water layers are set in other growth stages of rice. Therefore, in this context, rice is more likely to be directly affected by drought [32]. The regular occurrence of droughts during the rice-growing season is endangering food security.

Therefore, this study employs daily scale *SPEI* to quantitatively identify the short-term drought characteristics during the rice growth period in Heilongjiang Province from 2015 to 2100 under three scenarios: SSP1-2.6, SSP2-4.5 and SSP5-8.5. We aim to clarify the changing trends of *PET_0_* and *SPEI* during the rice growth period and reveal the meteorological drought characteristics during the rice growth period. This study will offer a scientific and reasonable basis for assessing the sensitivity of rice in Northeast China to future drought and provide a reasonable basis for the formulation of water resource management measures and regional water resource security.

## 2. Results

### 2.1. Temporal and Spatial Trends in Climatic Factors

**Figure 1:**
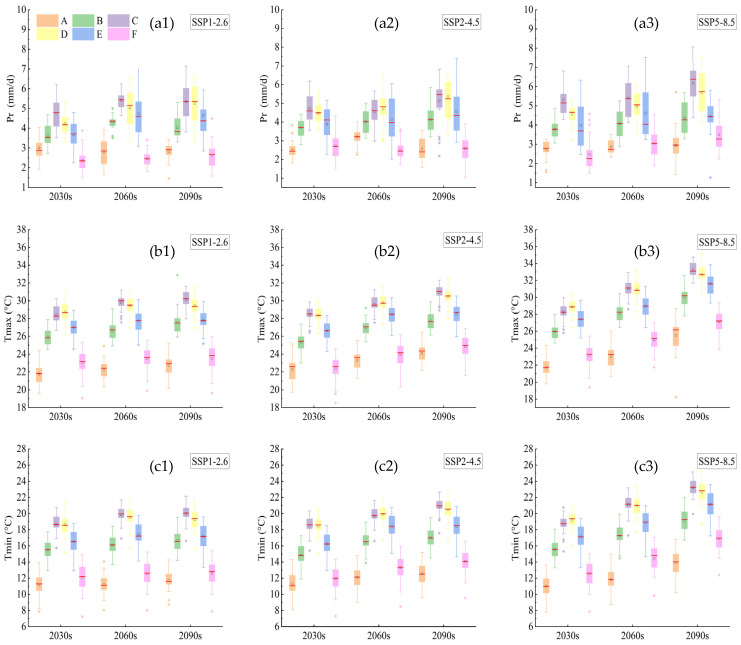
Trends of *P_r_* (**a1**–**a3**), *T_max_* (**b1**–**b3**) and *T_min_* (**c1**–**c3**) under SSP1-2.6, SSP2-4.5 and SSP5-8.5 during each growth period of rice from 2015 to 2100. *P_r_*, *T_max_* and *T_min_* represent precipitation, maximum air temperature and minimum air temperature, respectively. The 2030s, 2060s and 2090s represent the period of 2015–2040, 2041–2070 and 2071–2100, respectively. A, B, C, D, E and F represent the returning green stage, tillering stage, jointing booting stage, heading flower stage, milk stage and yellow ripening stage of rice, respectively. In the box-plot, the red line, the black square and the diamond represent the median, the mean and the outliers, respectively.

From the 2030s to the 2090s, *P_r_* exhibited an increasing trend from northwest to southeast (Figure S1(a1–a3,b1–b3,c1–c3)). The range of mean *P_r_* under SSP1-2.6, SSP2-4.5 and SSP5-8.5 were 3.61–4.12 mm/d, 3.66–4.05 mm/d, and 3.81–4.52 mm/d, respectively (Figure 1(a1–a3)). In both the B and D growth stages, *P_r_* showed an increasing trend from the 2030s to the 2090s under SSP2-4.5 and SSP5-8.5. Under the SSP2-4.5, *P_r_* in the A growth stage demonstrated a distinct trend of initially increasing and then decreasing from the 2030s to the 2090s (Figure 1(a2)). Under SSP5-8.5, *P_r_* in the C growth stage exhibited an increasing trend from the 2060s to the 2090s, with an increase of 0.7 mm/d (Figure 1(a3)).

*T_max_* and *T_min_* showed an increasing trend of from the northeast to the southwest from the 2030s to 2090s (Figures S2 and S3), the ranges of mean *T_max_* under SSP1-2.6, SSP2-4.5 and SSP5-8.5 was 25.82–26.87 °C, 25.58–27.76 °C and 25.9–30.04 °C, respectively (Figure 1(b1–b3)). Similarly, the mean values of *T_min_* under SSP1-2.6, SSP2-4.5 and SSP5-8.5 were 15.44–16.24 °C, 15.23–17.23 °C, and 15.68–19.48 °C, respectively (Figure 1(c1–c3)). The *T_max_* and *T_min_* in each growth stage exhibited an increased trend, with greater *T_max_* and Tmin in the C and D stages. In the 2090s, the *T_max_* and *T_min_* in stage C under the SSP5-8.5 reached their maximum temperature and minimum temperature of 32.5 °C and 23 °C, respectively (Figure 1(b1–b3,c1–c3)).

### 2.2. Spatial and Temporal Distribution and Trends of PET_0_

**Figure 2:**
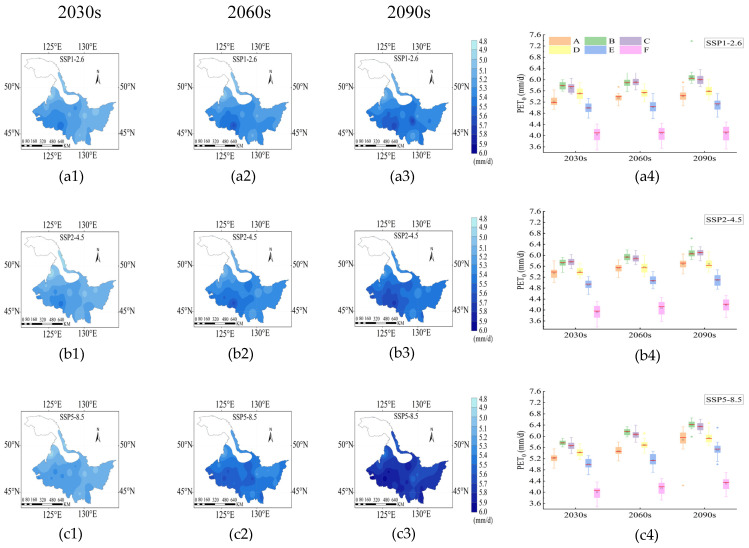
Spatiotemporal distribution of *PET_0_* under SSP1-2.6 (**a1**–**a4**), SSP2-4.5 (**b1**–**b4**) and SSP5-8.5 (**c1**–**c4**) during the rice growth period from 2015 to 2100. The 2030s, 2060s and 2090s represent the period of 2015–2040, 2041–2070 and 2071–2100, respectively. A, B, C, D, E and F represent the returning green stage, tillering stage, jointing booting stage, heading flower stage, milk stage and yellow ripening stage of rice, respectively. In the box-plot, the red line, the black square and the diamond represent the median, the mean and the outliers, respectively.

From the 2030s to 2090s, the high *PET_0_* was mainly concentrated in the southwest and central regions. Notably, the *PET_0_* under SSP5-8.5 was greater than those under SSP1-2.6 and SSP2-4.5 (Figure 2). The mean *PET_0_* under SSP1-2.6, SSP2-4.5 and SSP5-8.5 were 5.21–5.38 mm/d, 5.18–5.47 mm/d and 5.18–5.74 mm/d, respectively. Additionally, the *PET_0_* at each rice growth stage showed an increasing trend from the 2030s to the 2090s. The higher *PET_0_* were observed in the B and C stages, and in the B stage, the *PET_0_* reached 6.09 mm/d, 6.1 mm/d, and 6.4 mm/d under SSP1-2.6, SSP2-4.5, and SSP5-8.5 at 2090s, respectively. (Figure 2(a4–c4)).

### 2.3. Trend of SPEI

**Figure 3:**
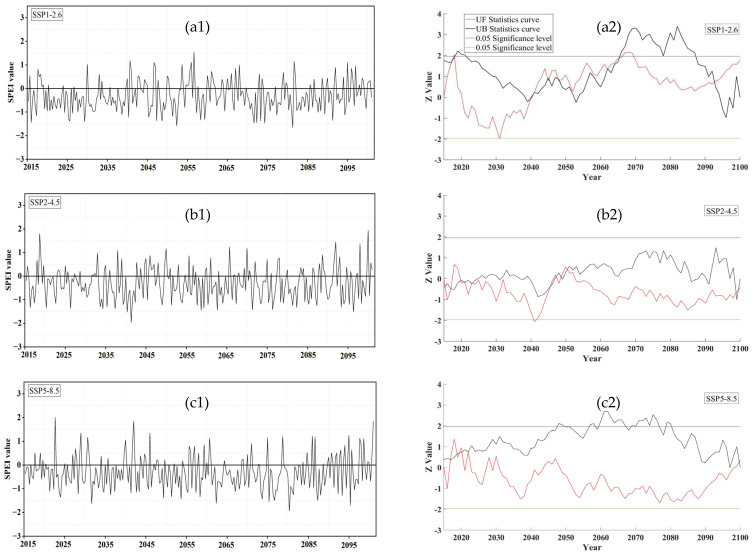
Changing trend in *SPEI* and Mann-Kendall mutation analysis under SSP1-2.6 (**a1**,**a2**), SSP2-4.5 (**b1**,**b2**) and SSP5-8.5 (**c1**,**c2**) during the rice growth period from 2015 to 2100.

From 2030s to 2090s, alternating wet and dry conditions were manifested. Evidently, the number of days with drought exceeded that of days without drought (Figure 3), and the daily mean *SPEI* ranged from −1.1 to 0.1, with the *SPEI* lower in the south region (Figure S4). Under SSP1-2.6, *SPEI* was mainly concentrated between no drought, mild drought and moderate drought (Figure 3(a1)), while under SSP2-4.5 and SSP5-8.5, *SPEI* was mainly concentrated between no drought, mild drought, moderate drought and severe drought (Figure 3(b1,c1)). Under both SSP1-2.6 and SSP5-8.5, *SPEI* showed a significant upward trend (Z > 1.96), with the *SPEI* mutation point interval ranging from 2017 to 2093 and 2017 to 2099, respectively (Figure 3(a2,c2)).

### 2.4. Drought Frequency (P_ij_)

**Figure 4:**
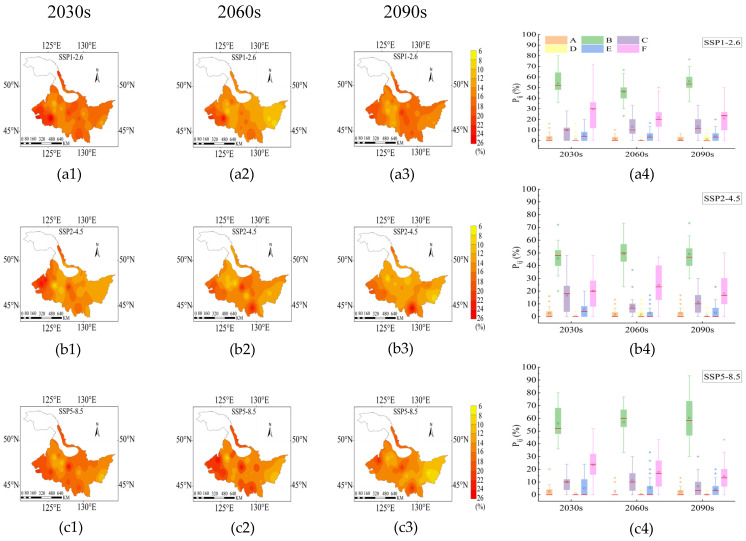
Distribution of *P_ij_* under SSP1-2.6 (**a1**–**a4**), SSP2-4.5 (**b1**–**b4**) and SSP5-8.5 (**c1**–**c4**) during the rice growth period from 2015 to 2100. The 2030s, 2060s and 2090s represent the period of 2015–2040, 2041–2070 and 2071–2100, respectively. A, B, C, D, E and F represent the returning green stage, tillering stage, jointing booting stage, heading flower stage, milk stage and yellow ripening stage of rice, respectively. In the box-plot, the red line, the black square and the diamond represent the median, the mean and the outliers, respectively.

From the 2030s to 2090s, the mean values of *P_ij_* during the rice growth period were 14.18–15.23%, 14.59–17.15% and 14.82–16.36%, respectively. The higher *P_ij_* was mainly concentrated in the western and southern regions (Figure 4). *P_ij_* showed a decreasing trend under SSP2-4.5 and SSP5-8.5 from 2030s to 2090s (Figure 4(b1–b3,c1–c3)). From the 2030s to the 2090s, *P_ij_* was highest in the B stage and lowest in the D stage (Figure 4(a4,b4,c4)). The drought frequency in the B and F stages fluctuated greatly, ranging from 63% to 72%, indicating a significant difference in the drought frequency between different stations (Figure 4(a4,b4,c4)).

### 2.5. Drought Duration (D_u_)

**Figure 5:**
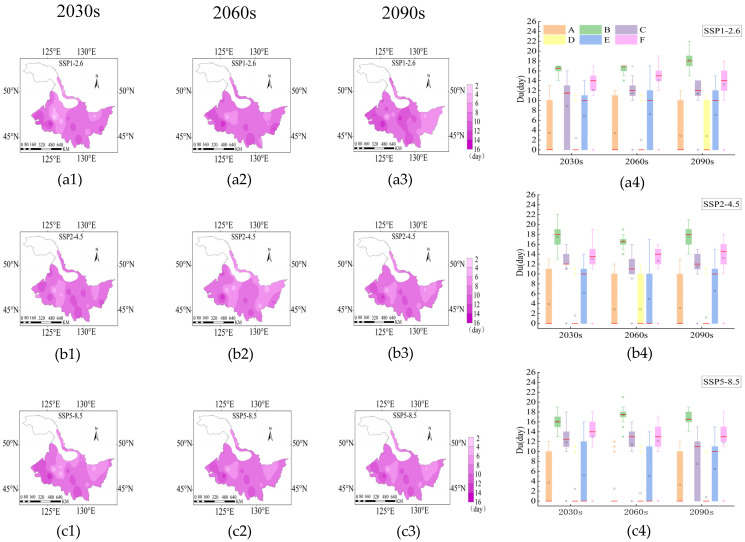
Distribution of *D_u_* under SSP1-2.6 (**a1**–**a4**), SSP2-4.5 (**b1**–**b4**) and SSP5-8.5 (**c1**–**c4**) during the rice growth period from 2015 to 2100. The 2030s, 2060s and 2090s represent the period of 2015–2040, 2041–2070 and 2071–2100, respectively. A, B, C, D, E and F represent the returning green stage, tillering stage, jointing booting stage, heading flower stage, milk stage and yellow ripening stage of rice, respectively. In the box-plot, the red line, the black square and the diamond represent the median, the mean and the outliers, respectively.

Under SSP1-2.6, SSP2-4.5 and SSP5-8.5 scenarios, the *D_u_* ranges 10–24, 10–22 and 10–21 days from 2030s to 2090s, respectively (Figure 5(a4,b4,c4)). The longest *D_u_* was mainly concentrated in the western and central parts of the study area (Figure 5(a1–a3,b1–b3,c1–c3). Rice experienced persistent drought conditions in stage B, and *D_u_* values in Fujin reached an extremely high value of 24 days in the 2060s under the SSP1-2.6 scenario (Figure 5(a4); Figure S5(a1)). Anda, Nenjiang, Qiqihar, Tieli, Tonghe, and Mudanjiang, located in the west, south and center regions, suffered from persistent droughts under the SSP2-4.5 and SSP5-8.5, and droughts occurred with the highest frequency at B, C, E and F stages. While *D_u_* at the D stage in most regions was 0 (Figure S5).

### 2.6. Drought Intensity (IN) and Drought Barycenter

**Figure 6:**
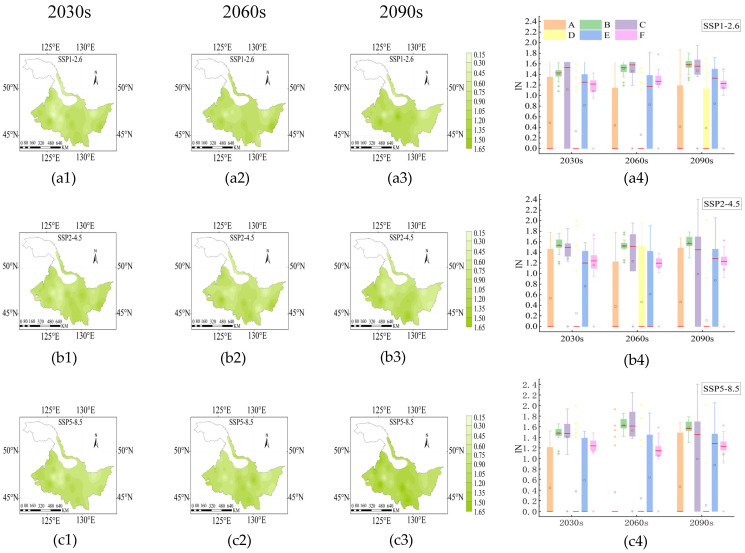
Distribution of *IN* under SSP1-2.6 (**a1**–**a4**), SSP2-4.5 (**b1**–**b4**) and SSP5-8.5 (**c1**–**c4**) during the rice growth period from 2015 to 2100. The 2030s, 2060s and 2090s represent the period of 2015–2040, 2041–2070 and 2071–2100, respectively. A, B, C, D, E and F represent the returning green stage, tillering stage, jointing booting stage, heading flower stage, milk stage and yellow ripening stage of rice, respectively. In the box-plot, the red line, the black square and the diamond represent the median, the mean and the outliers, respectively.

**Figure 7:**
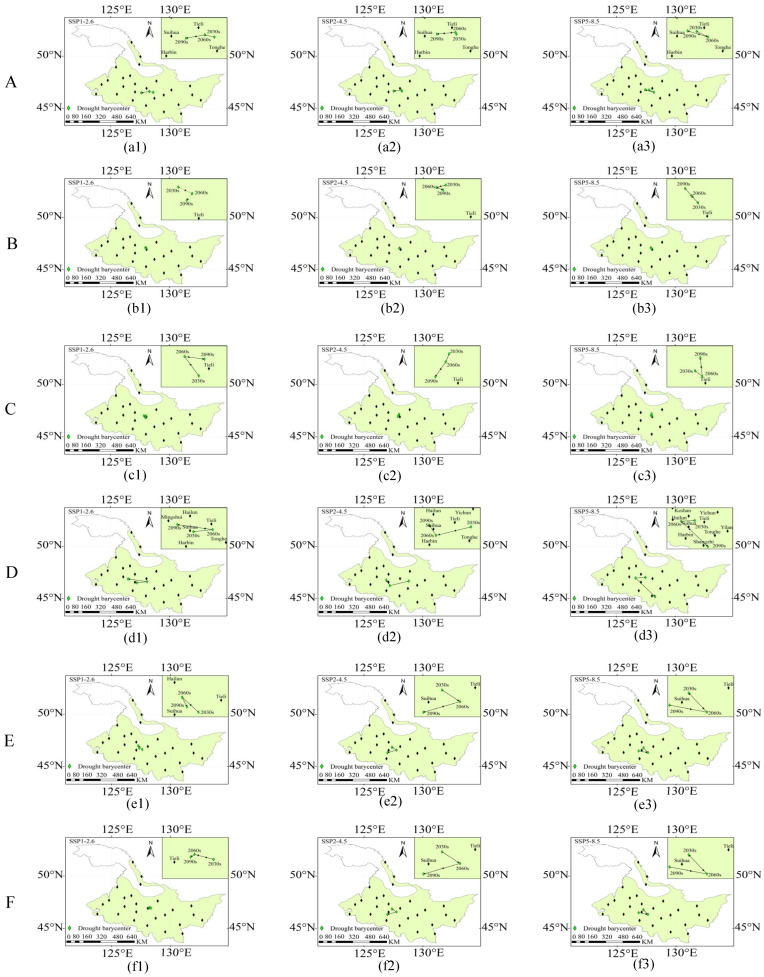
The trajectory of the drought barycenter under SSP1-2.6 (**a1**–**f1**), SSP2-4.5 (**a2**–**f2**) and SSP5-8.5 (**a3**–**f3**) during each growth period of rice from 2015 to 2100. A, B, C, D, E and F represent the returning green stage, tillering stage, jointing booting stage, heading flower stage, milk stage and yellow ripening stage of rice, respectively.

From 2030s to 2090s, the mean values of *IN* during the rice growth period were 0.87–0.97, 0.89–0.92, and 0.85–0.91 under SSP1-2.6, SSP2-4.5, and SSP5-8.5 (Figure 6), with the high *IN* areas concentrated in the northwest and central regions (Figure 6(a1–a3,b1–b3,c1–c3)). Stage B and D had the highest and lowest *IN*, respectively (Figure 6(a4,b4,c4)). The drought barycenter was mainly distributed around Tieli and Suihua (Figure 7), and the trajectory of the drought barycenter in the D stage migrated from Tieli in the central part to Suihua and Mingshui areas in the west under the SSP1-2.6 and SSP2-4.5 (Figure 7(d1,d2)). Notably, the drought barycenter of the D stage migrated from west to south from the 2060s to 2090s under the SSP5-8.5 scenario (Figure 7(d3)).

### 2.7. Correlation of Meteorological Factors with PET_0_ and SPEI

From the 2030s to 2090s, significant positive correlations between *PET_0_* and *T_max_*, *T_min_* were observed under SSP1-2.6, SSP2-4.5 and SSP5-8.5 (Table S2). Additionally, at B, C, D and F stages, *PET_0_* demonstrated a significant positive correlation with *P_r_*. The correlation coefficients of *SPEI* with *T_max_* and *T_min_* were greater than *P_r_*, indicating that air temperature had a stronger effect on *PET_0_* than *P_r_*.

Under SSP2-4.5 and SSP5-8.5, *SPEI* was significantly positively correlated with *P_r_* and negatively correlated with *T_max_* and *T_min_* at the A stage. Under scenarios SSP1-2.6 and SSP5-8.5, *SPEI* was significantly positively correlated with *P_r_* at the B and F stages. Under scenario SSP5-8.5, *SPEI* was significantly positively correlated with *P_r_*, *T_max_*, and *T_min_* at the C stage. The correlation coefficients between *SPEI* and *P_r_* were greater than *T_max_* and *T_min_*, indicating that *P_r_* had a greater effect on *SPEI* than air temperature.

## 3. Discussion

Temperature is recognized as a key factor influencing *PET_0_* increase in Bangladesh from 2061 to 2099 [33]. Similarly, Guo et al. revealed that temperature was the primary factor affecting *PET_0_* increase in Australia from 1995 to 2004 [34]. It is crucial to investigate how temperature increases brought on by climate change affect the emergence of droughts, according to Gumus [35]. Research on the Sanjiang Plain and this study both showed that rising temperatures had a greater impact on *PET_0_* than increases in precipitation, with the greatest temperature serving as the primary driver of *PET_0_* growth during the rice growth period [10]. The importance of temperature in escalating drought conditions, particularly in arid and semi-arid regions, was highlighted by Ahmed et al. [36]. This implies that global warming may worsen drought and make water security increasingly challenging. Temperature plays an essential part in the development of drought during the rice-growing season. Figure 2 and Figure 3 also indicate that irrespective of trends in precipitation, rising temperatures increase evapotranspiration and transpiration, which raises the need for water for activities including agriculture and ultimately exacerbates the risk of drought [37].

The causes of spatial and temporal evolution of drought are closely related to land-air interactions [38]. Meteorological factors, as the most fundamental factors, control the formation of climate [39]. A positive feedback process between the land and atmosphere intensifies meteorological drought when it is accompanied by high temperatures and heat waves. Short-term droughts were more frequent in the southern region of Northeast China, and high temperatures may be the primary cause of their occurrence, according to the findings of Xue et al.’s study on the spatial and temporal characteristics and drivers of sudden droughts in northern China from 1978 to 2020 [40]. In our study, although Mudanjiang and other southern regions have high drought frequency, high drought intensity, and long drought duration, they have sufficient precipitation, low temperatures, and lower *PET_0_*. This condition could arise from the extreme variations in temperature and precipitation in these locations, which might also be impacted by air circulation and monsoons, with a clear regional pattern of drought [41,42].

Short-term droughts are characterized by strong flashbacks, rapid development, high intensity, and difficulty forecasting compared with traditional droughts [43]. An annual scale *SPEI* analysis by Mei et al. revealed that Heilongjiang Province’s drought characteristics from 2000 to 2018 were mostly defined by an east-west pattern of wet and dry in space, with no drought in the summer and moderate drought in autumn [44]. In contrast, the results of our study based on daily-scale *SPEI* showed that moderate and severe drought will occur during the rice growing period; this suggests that the daily-scale *SPEI* may be able to detect more severe potential drought. Especially in the southern regions such as Mudanjiang and Suifenhe, the *SPEI* values during the rice growing period ranged from −1.5 to −0.5 (Figure S4(a1–a3,b1–b3,c1–c3)), with moderate drought. Li et al. indicated that southern regions in Heilongjiang Province, such as Mudanjiang, had *SPEI* values ranging from −2.0–−1.7 with severe drought over the 2011–2020 time period [45]. As a result, in the future, Heilongjiang Province’s southern region will experience less drought.

This study quantitatively identified the droughts at each growth stage of rice under future scenarios. According to our study, the tillering stage of rice growth has the highest frequency, duration, and intensity of droughts. The main cause of drought occurrence was found to be *PET_0_*, which is higher than precipitation. (Figure 1(a1–a3) and Figure 2(a4,b4,c4)). Prolonged drought during the tillering stage might hinder rice development at the following four growth stages, lowering rice yield [45]. The ability of early warning services should be strengthened to prevent such drought occurrences, along with strategies like rational irrigation [46], modifying the planting and harvesting dates [47], and other efficient drought resistance and disaster reduction methods [42]. The heading stage is characterized by the lowest drought intensity and the most volatility, particularly in the 2090s under SSP1-2.6 and 2060s under SSP2-4.5 (Figure 6(a4,b4)). This variation could be caused by the extreme precipitation of an abrupt rise in precipitation from the 2060s to the 2090s (Figure 1(a1,a2)). The excessive precipitation conditions might also delay rice growth and lower yields [29]. Furthermore, the migration of the drought barycenter may result from variations in precipitation in different regions. The trajectories of the drought barycenter under the SSP1-2.6, SSP2-4.5, and SSP5-8.5 scenarios varied greatly between the 2030s and 2090s at the heading flower stage (Figure 7(d1–d3), indicating variability and fluctuation in drought trajectory characteristics across different SSP scenarios. The severe lack of precipitation and the greater *PET_0_* in the western section of Heilongjiang Province may have caused the drought barycenter to migrate from the central to the western part of the province in the 2060s and 2090s (Figures S1 and S2 and Figure 2). Ng et al. revealed that the drought barycenter in Peninsular Malaysia shifted from the central to the northern and southern regions, and this shift was also attributed to the lack of precipitation in the northern and southern parts [48]. The sudden migration of drought barycenter from Hailun to around Shangzhi from the 2060s to 2090s under the SSP5-8.5 scenario (Figure 7(d3)) might be attributed to the increase in drought intensity and drought frequency around the region (Figure 5 and Figure 6). To alleviate localized drought situations, water supplies in the southeast can be rationally allocated to water-scarce areas in the northwest [49].

In this study, considerable alterations in drought types were caused by short-term drought. For instance, in 2040, the *SPEI* mutation point under the SSP1-2.6 scenario, the drought type changed from mild drought to no drought, and the *SPEI* trend changed from declining to increasing (Figure 3(a1,a2)). Under the SSP2-4.5 scenario from 2015 to 2064, *SPEI* first exhibited a major fall, followed by a slight increase, and then another significant decrease (Figure 3(b1,b2)), which caused the drought to intensify from mild drought to moderate drought, thus increasing the severity of drought. *SPEI* is not sensitive enough to capture extreme drought events in China, although it can generally record large drought events [50]. Research on extreme drought in agriculture should be investigated in the future; this might require a comprehensive assessment of multiple drought indicators.

## 4. Materials and Methods

### 4.1. Study Area, Data Sources and Rice Growth Period Division

**Figure 8:**
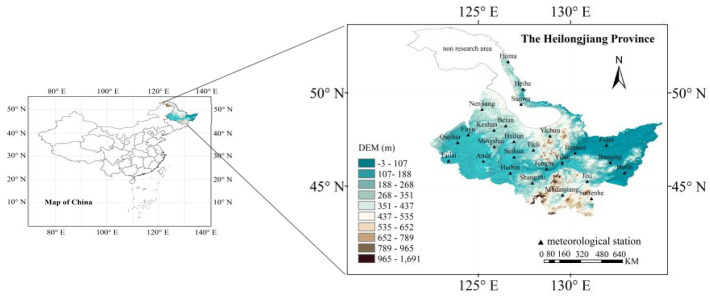
Overview of the study area.

The study area is located in Heilongjiang Province in Northeastern China, lies between latitudes 43°26′–53°33′ N and longitudes 121°11′–135°05′ E, with a total jurisdictional area of 473 million km^2^, the 6th largest in China. Heilongjiang Province belongs to the continental monsoon climate of cold temperate and temperate zones. It can be divided into humid, semi-humid, and semi-arid zones based on dryness indicators from east to west. The average annual temperature is −5–5 °C, and the annual precipitation is between 400 and 800 mm. Figure 8 provides an overview of the study area, while the sixth accumulated temperature zone, which is currently unsuitable for rice production, is represented by the blank area.

Meteorological data were obtained from the NorESM2-MM model in CMIP6 with a resolution of 2.5° × 1.875°. We used the bilinear interpolation method on the statistical downscaling scale to obtain the meteorological datasets of daily precipitation (*P_r_*), maximum air temperature (*T_max_*) and minimum air temperature (*T_min_*) for three future time periods (2015–2040, 2041–2070 and 2071–2100, defined as 2030s, 2060s and 2090s) under SSP1-2.6, SSP2-4.5 and SSP5-8.5 at 26 meteorological stations in Heilongjiang Province.

The data for the rice growth period emerged from the “Experimental Study on Irrigation of Rice and Evaluation of Irrigation in Zones in Heilongjiang Province” (Table S1). The rice growth periods were classified as follows: returning green stage (A), tillering stage (B), jointing booting stage (C), heading flower stage (D), milk stage (E) and yellow ripening stage (F) [51]. We assumed that the length of each rice growth period would not change in 3 future time periods [52].

### 4.2. Standardized Precipitation Evapotranspiration Index (SPEI)

The daily *PET_0_* is calculated using the FAO-recommended Hargreaves method [53],

(1) $${{PET}_{0}}^{\prime }=0.408\times \alpha \times Ra(T_{mean}+17.8){\left(T_{\max}-T_{\min}\right)}^{0.5}$$

where *PET_0′_* is the potential equivalent evapotranspiration calculated by empirical formula (mm/d), α is a constant, 0.0023; *T_max_* and *T_min_* are the daily maximum and minimum air temperatures (°C), respectively, *T_mean_* is the daily average temperature (°C), *R_a_* is the zenith radiation (MJm^−2^ day^−1^), calculated using reference [54].

Since Equation (1) is obtained from an empirical formula, ref. [55] adjusted *PET_0′_* by regression analysis. The fitted *PET_0_* is as follows:

(2) $${PET}_{0}=a+{{bPET}_{0}}^{\prime }$$

where *PET_0_* is the adjusted *PET_0′_* (mm/d), *a* and *b* are correction coefficients, which are constants at a particular spatiotemporal scale.

Cumulative moisture deficit (*D_i_*):

(3) $$D_{i}=P_{i}-({PET}_{0}{)}_{i}$$

where *D_i_* is the cumulative moisture deficit (mm/d), *P_i_* is the daily precipitation (mm/d); (*PET_0_*)_i_ is the daily *PET_0_* (mm/d).

The three-parameter Log-Logistic distribution of the probability density function was used to describe the water deficit time series, and its probability density function *f*(*x*) and probability distribution function *F*(*x*) are calculated as follows:

(4) $$f(x)=\frac{\beta }{\alpha }(\frac{x-\gamma }{\alpha }){\left[1+{\left(\frac{x-\gamma }{\alpha }\right)}^{\beta }\right]}^{-2}$$

where *α* is the scale parameter; *β* is the shape parameter; *γ* is the position parameter; the range of *D_i_* value is (*γ*,∞). Where *α*, *β* and *γ* can be fitted by the linear moment method, the calculation formula is as follows:

(5) $$\beta =\frac{2w_{1}-w_{0}}{6w_{1}-w_{0}-6w_{2}}$$

(6) $$\alpha =\frac{\beta (w_{0}-2w_{1})}{\Gamma (1+1/\beta )\Gamma (1-1/\beta )}$$

(7) $$\gamma =w_{0}-\alpha \Gamma (1+1/\beta )\Gamma (1-1/\beta )$$

where Γ(*β*) is the Gamma distribution function of (*β*); ω_s_ is the probability-weighted moment of *D_i_* (*s* = 0, 1, 2), which is calculated as follows:

(8) $$w_{s}=\frac{1}{n}{\sum_{i}^{n}\left(\frac{j-0.35}{n}\right)}^{s}D_{i}$$

*P*(*D*) is the probability of being greater than *D* for a given daily scale, which is given by:

(9) P(D)=1−F(x)

When *P*(*D*) ≤ 0.5, the *SPEI* is calculated using Equation (10), while when *P*(*D*) > 0.5, the *SPEI* is calculated using Equation (11).

(10) $$SPEI=w-\frac{\left(c_{0}+c_{1}w+c_{2}w^{2}\right)}{1+d_{1}w+d_{2}w^{2}+d_{3}w^{3}},w=\sqrt{-2\ln P(D)}$$

(11) $$SPEI=-(w-\frac{c_{0}+c_{1}w+c_{2}w^{2}}{1+d_{1}w+d_{2}w^{2}+d_{3}w^{3}}),w=\sqrt{-2\ln[1-P(D)]}$$

where *c*_0_ = 2.515517, *c*_1_ = 0.802853, *c*_2_ = 0.010328, *d*_1_ = 1.432788, *d*_2_ = 0.189269, *d*_3_ = 0.001308 [56].

Table 1 presents the drought classification based on *SPEI* according to the meteorological drought classification issued by the National Climate Center [57].

### 4.3. Drought Characteristics

Drought characteristics include drought frequency, drought duration, drought intensity and drought barycenter.

Drought frequency (*P_ij_*) describes the proportion of years with drought compared to the total number of years. This study calculates the drought frequency based on the *SPEI* of each meteorological station.

(12) $$P_{\mathrm{ij}}=\frac{n_{\mathrm{ij}}}{N}\times 100\%$$

where *n_ij_* is the number of years in which drought of level *j* occurred during the i growth period, and N is the total number of years. The larger the value of *P_ij_*, the higher the frequency of drought occurrence.

A drought process was deemed to have taken place when the *SPEI* remained less than or equal to −0.5 for 10 successive days. The initial day within these 10 consecutive days marks the commencement of the drought process. Once the *SPEI* exceeds −0.5 for another 10 consecutive days, the drought process is then considered to have terminated. The day preceding the first day of those 10 days is regarded as the end date of this particular drought event. The time interval stretching from the onset to the conclusion of the drought process forms the drought duration (*D_u_*) [1].

Drought intensity (*IN*) is calculated as the ratio of the cumulative *SPEI* for all days of the drought process above mild drought to the duration of the drought event, with larger values indicating a more intense drought process.

(13) $$IN=\frac{1}{n}|\sum_{i=1}^{n}{\left(\mathrm{SPEI}\right)}_{i}|\;$$

where n represents the number of days the drought event lasted.

The drought barycenter is proposed based on the barycenter theory, which is expressed as the location of drought events in longitude, latitude, and time. The formula for calculating the drought barycenter in this study is as follows:

(14) $$X_{i}=\frac{\sum_{s=1}^{n}W_{i,\;s}X_{s}}{\sum_{s=1}^{n}W_{i,\;s}},Y_{i}=\frac{\sum_{s=1}^{n}W_{i,\;s}Y_{s}}{\sum_{s=1}^{n}W_{i,\;s}}$$

where *X_i_* and *Y_i_* represent the latitude and longitude of the regional barycenter of drought during the *i*-growth period (°), respectively; *W_i,s_* represent the drought intensity of the sth meteorological station in the *i*-growth period; *X_s_* and Y_s_ represent the latitude and longitude of the sth meteorological station (°).

### 4.4. Pearson Correlation Coefficient

The Pearson correlation coefficient quantitatively describes the degree of correlation between climatic factors and drought characteristics [58]. If there are n data points (x*_i_*,y*_i_*) (*i* = 1, 2,…, n), the formula is as follows:

(15) $$r=\frac{\sum_{i=0}^{n}\left(x_{i}-\overset{-}{x})(y-\overset{-}{y}\right)}{\sqrt{\sum_{i=0}^{n}{\left(x_{i}-\overset{-}{x}\right)}^{2}\sum_{i=0}^{n}{\left(y-\overset{-}{y}\right)}^{2}}}$$

In the formula, the value of *r* ranges from −1 to 1, where −1 and 1 represent positive and negative correlation, respectively, while 0 represents no correlation, the closer |*r*| is to 0, the weaker the correlation is, and the closer |*r*| is to 1, the stronger the correlation is [1].

### 4.5. Mann-Kendall Trend Test

The Mann-Kendall trend test, which was introduced by the World Meteorological Organization, is a non-parametric statistical approach utilized to uncover the manner in which a variable changes over time. The positive and negative values of the statistical variable *Z* signify the trend of the data.

In general, when the significance level α is set to 0.05, the critical value of *Z* is ±1.96. The curves of the two statistical series, namely UF and UB, along with the two straight lines corresponding to ±1.96, are plotted on a single graph. For detailed analysis, references [59,60] can be consulted.

### 4.6. Data Processing

We extracted the *P_r_*, *T_max_* and *T_min_* datasets from the CMIP6 by employing the bilinear interpolation statistical downscaling method (https://aims2.llnl.gov/search/cmip6/ (accessed on 1 February 2023)). The *PET_0_*, drought frequency, drought duration, and drought intensity were computed by VBA macros 7.1(Microsoft Corporation, Redmond, WA, USA), *SPEI* and its Mann -Kendall trend test were calculated and performed by MATLAB R2020b (MathWorks, Natick, MA, USA). Pearson correlation analysis of *P_r_*, *T_max_*, *T_min_*, *PET_0_* and *SPEI* was conducted using SPSS27.0 (IBM Corporation, Armonk, NY, USA). Drought ephemeral distribution heat maps and box plots were plotted using Origin 2021 (OriginLab Inc., Northampton, MA, USA). The spatiotemporal distribution map of drought features was generated by the inverse distance weight method (IDW) in ArcMap 10.7 (Environmental Systems Institute, Redlands, CA, USA)

## 5. Conclusions

This study explored the temporal and spatial patterns of drought characteristics and their driving factors under different SSP1-2.6, SSP2-4.5, and SSP5-8.5 from 2015 to 2100. *SPEI* showed a decreasing trend sequentially from the northeast to the southwest, and *P_r_* had the most impact on *SPEI*. *PET_0_* at each rice growth stage showed an increasing trend from the 2030s to 2090s, with the largest *PET_0_* at the tillering stage and the lowest *PET_0_* at the yellow ripening stage. Additionally, *PET_0_* was most affected by *T_max_*. The tillering stage experienced the highest drought frequency and drought intensity covering the whole study area, with the longest drought duration being 24 days. While the heading flower stage experienced the highest precipitation, lowest drought frequency, and lowest drought intensity. Developing more accurate and efficient drought monitoring and early warning systems, as well as promoting the implementation of sustainable water resource management strategies, will help better address the challenges of short-term drought and ensure the sustainable development of agriculture and water resources.

## Figures and Tables

**Table 1:** Drought classification based on *SPEI*.

Drought Classification	*SPEI*
No drought	*SPEI* > −0.5
Mild drought	−0.5 ≥ *SPEI* > −1.0
Moderate drought	−1.0 ≥ *SPEI* > −1.5
Severe drought	−1.5 ≥ *SPEI* > −2.0
Extreme drought	*SPEI* ≤ −2.0

## Data Availability

Data that support the findings of this study are available from the corresponding author upon reasonable request.

## Supplementary Materials

The following supporting information can be downloaded at: https://www.mdpi.com/article/10.3390/plants14010030/s1, Figure S1. Spatial and temporal distribution of *P_r_* under SSP1-2.6 (a1–a3), SSP2-4.5 (b1–b3) and SSP5-8.5 (c1–c3) during rice growth period from 2015 to 2100; Figure S2. Spatial and temporal distribution of *T_max_* under SSP1-2.6 (a1–a3), SSP2-4.5 (b1–b3) and SSP5-8.5 (c1–c3) during rice growth period from 2015 to 2100; Figure S3. Spatial and temporal distribution of *T_min_* under SSP1-2.6 (a1–a3), SSP2-4.5 (b1–b3) and SSP5-8.5 (c1–c3) during rice growth period from 2015 to 2100; Figure S4. Spatial and temporal distribution of *SPEI* under SSP1-2.6 (a1–a3), SSP2-4.5 (b1–b3) and SSP5-8.5 (c1–c3) during rice growth period from 2015 to 2100; Figure S5. Distribution of *D_u_* under SSP1-2.6 (a1), SSP2-4.5 (a2) and SSP5-8.5 (a3) during the rice growth period from 2015 to 2100. The 2030s, 2060s and 2090s represent the period of 2015–2040, 2041–2070 and 2071–2100, respectively. A, B, C, D, E and F represent the returning green stage, tillering stage, jointing booting stage, heading flower stage, milk stage and yellow ripening stage of rice, respectively. Table S1. Duration of the growth period of rice in 26 selected areas in Heilongjiang Province; Table S2. Correlation of *PET_0_* and *SPEI* with *T_max_*, *T_min_* and *P_r_* during the rice growth period.
