# Supplementary material for: Characterizing Droughts During the Rice Growth Period in Northeast China Based on Daily SPEI Under Climate Change
Source: Plants (Basel). 2024 Dec 25;14(1):30. doi: 10.3390/plants14010030 (PMC11723174; doi:10.3390/plants14010030)
Supplement: Supplementary file 1 [file plants-14-00030-s001.zip › plants-3261621-supplementary/Supplementary Files/supplementary figures.pdf]

Figure captions

Fig.S1.Spatial and temporal distribution of P_r under SSP1-2.6 (a1-a3), SSP2-4.5 (b1-b3) and SSP5-8.5 (c1-c3) during rice growth period from 2015 to 2100. 2030s, 2060s and 2090s represent the period of 2015-2040, 2041-2070 and 2071-2100, respectively.

Fig.S2. Spatial and temporal distribution of T_{max} under SSP1-2.6 (a1-a3), SSP2-4.5 (b1-b3) and SSP5-8.5 (c1-c3) during rice growth period from 2015 to 2100. 2030s, 2060s and 2090s represent the period of 2015-2040, 2041-2070 and 2071-2100, respectively.

Fig.S3. Spatial and temporal distribution of T_{min} under SSP1-2.6 (a1-a3), SSP2-4.5 (b1-b3) and SSP5-8.5 (c1-c3) during rice growth period from 2015 to 2100. 2030s, 2060s and 2090s represent the period of 2015-2040, 2041-2070 and 2071-2100, respectively.

Fig.S4. Spatial and temporal distribution of *SPEI* under SSP1-2.6 (a1-a3), SSP2-4.5 (b1-b3) and SSP5-8.5 (c1-c3) during rice growth period from 2015 to 2100. 2030s, 2060s and 2090s represent the period of 2015-2040, 2041-2070 and 2071-2100, respectively.

Fig.S5. Distribution of D_u under SSP1-2.6 (a1), SSP2-4.5 (a2) and SSP5-8.5 (a3) during the rice growth period from 2015 to 2100. 2030s, 2060s and 2090s represent the period of 2015-2040, 2041-2070 and 2071-2100, respectively. A, B, C, D, E and F represent returning green stage, tillering stage, jointing booting stage, heading flower stage, milk stage and yellow ripening stage of rice, respectively

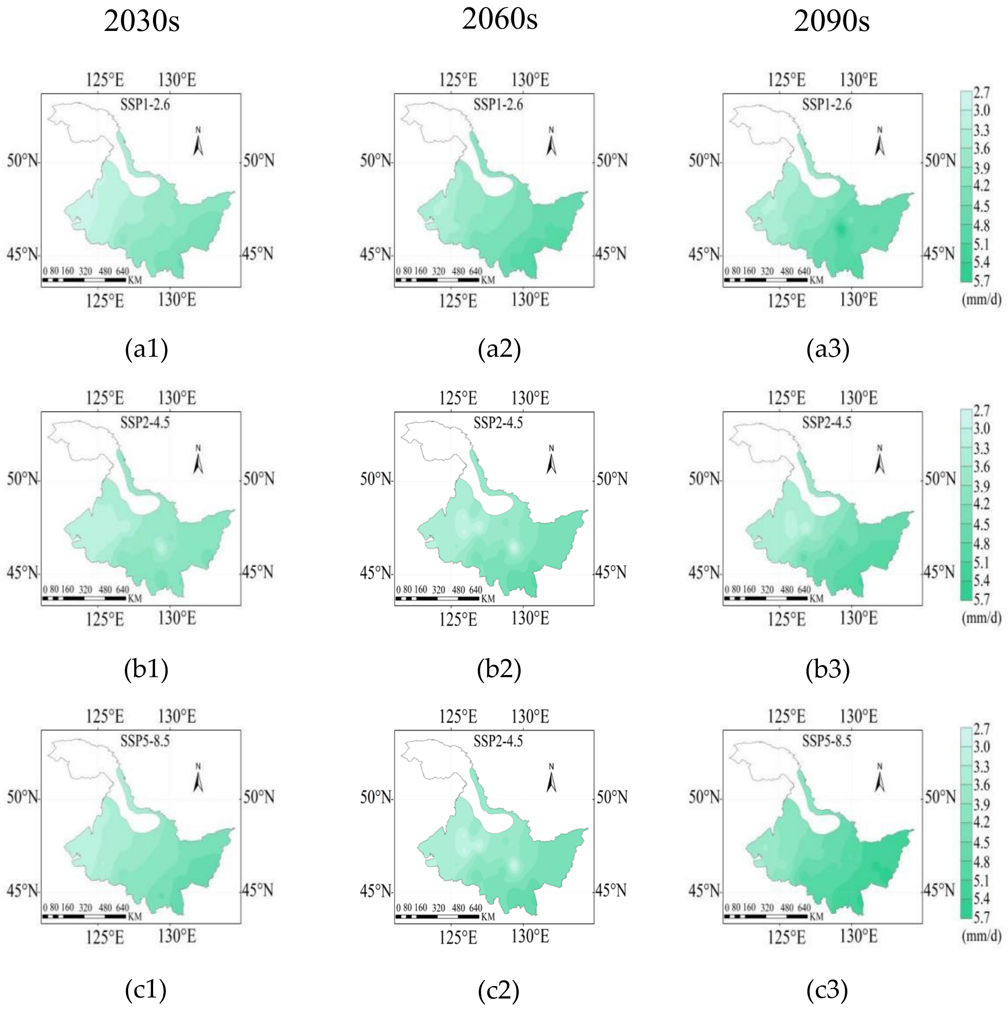

Figure S1.Spatial and temporal distribution of P_r under SSP1-2.6 (a1-a3), SSP2-4.5 (b1-b3) and SSP5-8.5 (c1-c3) during rice growth period from 2015 to 2100. 2030s, 2060s and 2090s represent the period of 2015-2040, 2041-2070 and 2071-2100, respectively.

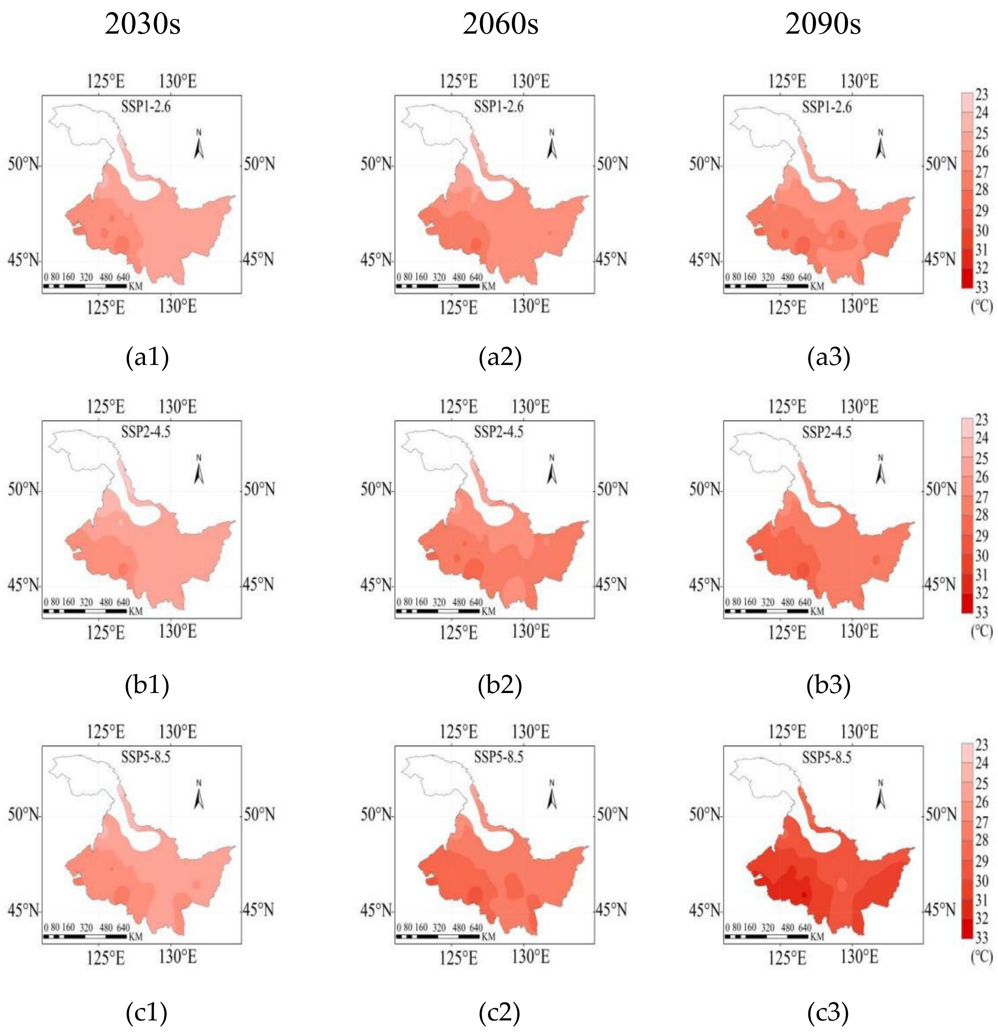

Figure S2. Spatial and temporal distribution of T_{max} under SSP1-2.6 (a1-a3), SSP2-4.5 (b1-b3) and SSP5-8.5 (c1-c3) during rice growth period from 2015 to 2100. 2030s, 2060s and 2090s represent the period of 2015-2040, 2041-2070 and 2071-2100, respectively.

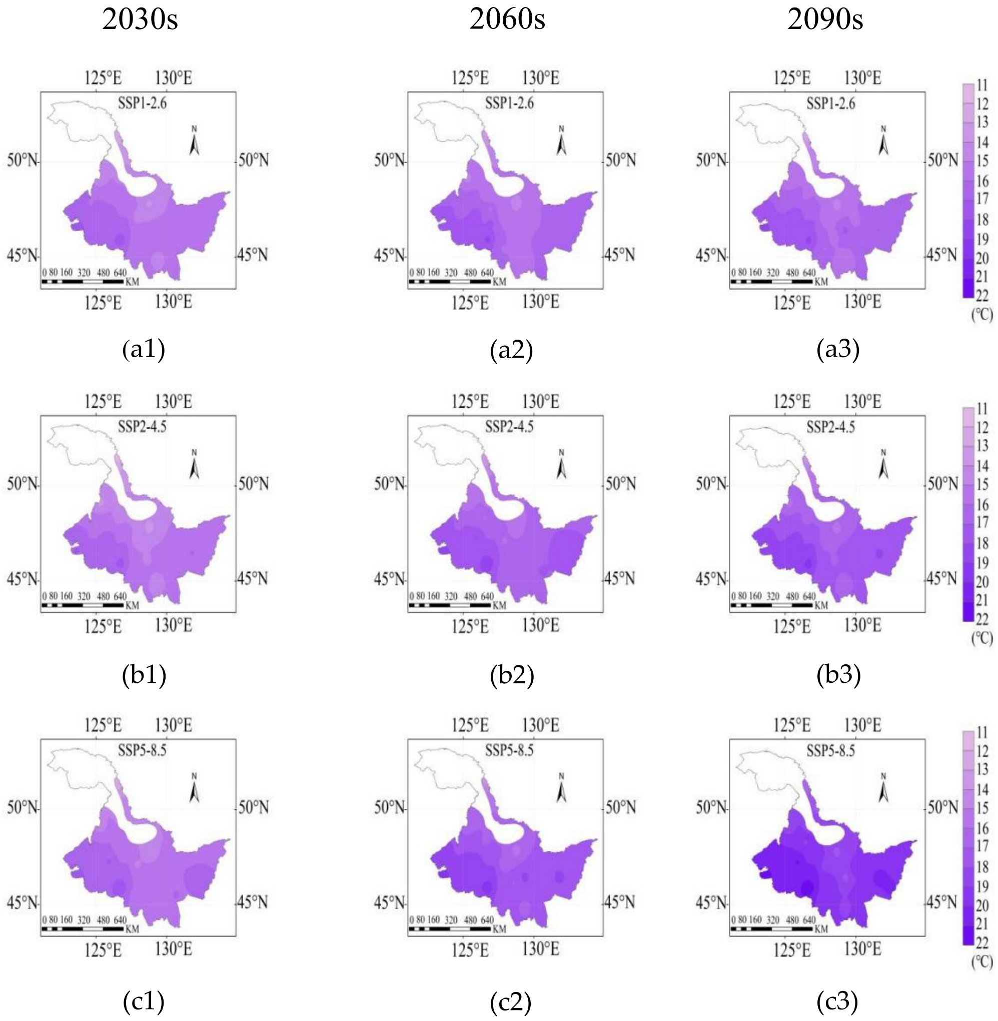

Figure S3. Spatial and temporal distribution of T_{min} under SSP1-2.6 (a1-a3), SSP2-4.5 (b1-b3) and SSP5-8.5 (c1-c3) during rice growth period from 2015 to 2100. 2030s, 2060s and 2090s represent the period of 2015-2040, 2041-2070 and 2071-2100, respectively.

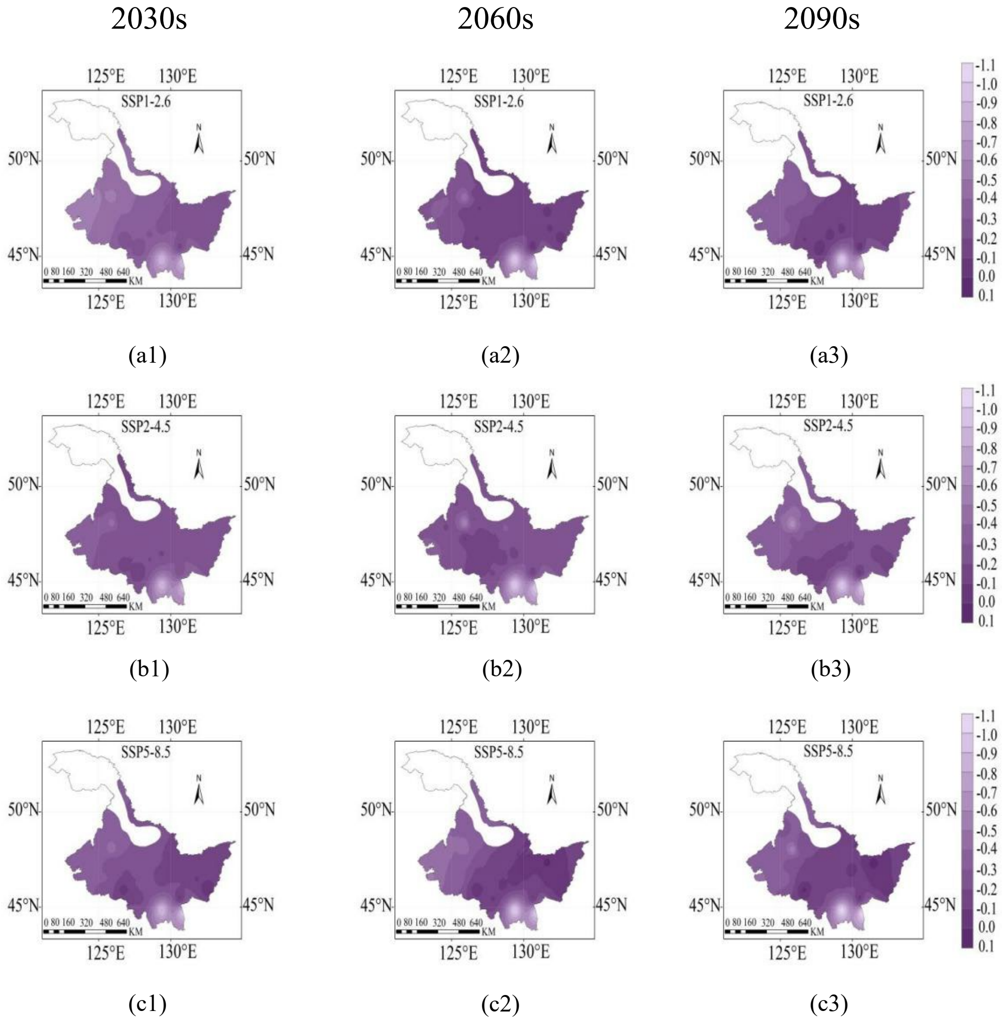

Figure S4. Spatial and temporal distribution of *SPEI* under SSP1-2.6 (a1-a3), SSP2-4.5 (b1-b3) and SSP5-8.5 (c1-c3) during rice growth period from 2015 to 2100. 2030s, 2060s and 2090s represent the period of 2015-2040, 2041-2070 and 2071-2100, respectively.

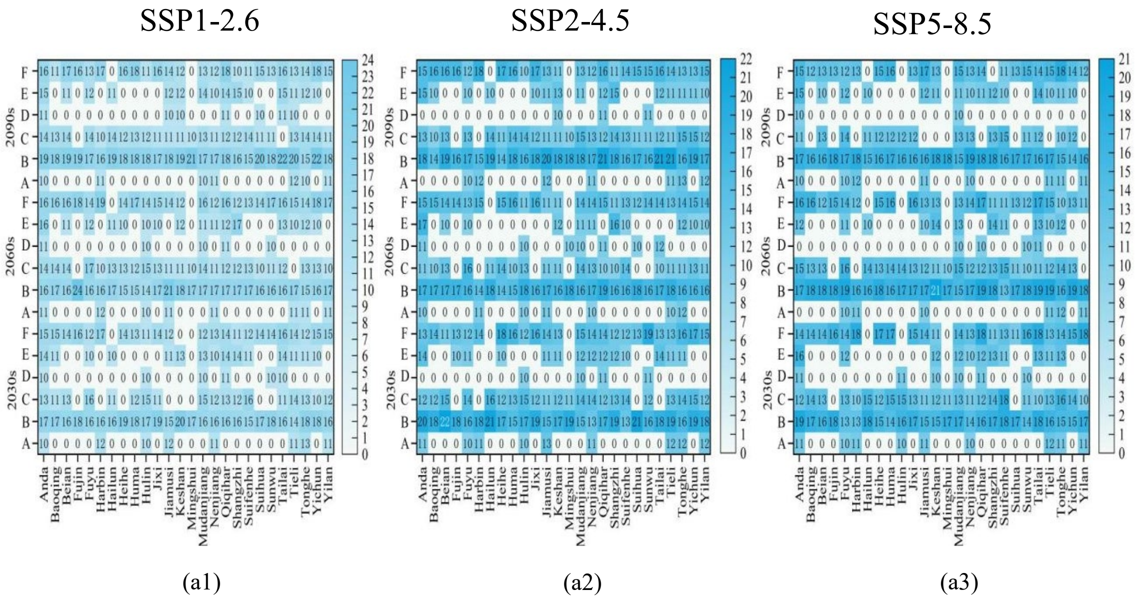

Figure S5. Distribution of D_u under SSP1-2.6 (a1), SSP2-4.5 (a2) and SSP5-8.5 (a3) during the rice growth period from 2015 to 2100. 2030s, 2060s and 2090s represent the period of 2015-2040, 2041-2070 and 2071-2100, respectively. A, B, C, D, E and F represent returning green stage, tillering stage, jointing booting stage, heading flower stage, milk stage and yellow ripening stage of rice, respectively
