# Supplementary material for: Characterizing Droughts During the Rice Growth Period in Northeast China Based on Daily SPEI Under Climate Change
Source: Plants (Basel). 2024 Dec 25;14(1):30. doi: 10.3390/plants14010030 (PMC11723174; doi:10.3390/plants14010030)
Supplement: Supplementary file 1 [file plants-14-00030-s001.zip › plants-3261621-supplementary/Supplementary Files/supplementary tables.pdf]

Table captions

Table S1. Duration of the growth period of rice in 26 selected areas in Heilongjiang Province.

Table S2. Correlation of PET_0 and $SPEI$ with T_{max}, T_{min} and P_r during the rice growth period.

Table S1. Duration of the growth period of rice in 26 selected areas in Heilongjiang Province.

Meteorologi-cal station	Returning green stage(days)	Tillering stage (days)	Jointing booting stage(days)	Heading flower stage (days)	Milk stage (days)	Yellow ripening stage(days)	Total Growth day□ days □
Anda	12	34	19	14	22	24	125
Fuyu	11	38	20	9	17	19	114
Lailun							
Beian	9	34	22	10	13	25	113
Yichun							
Keshan	7	35	14	12	16	19	103
Sunwu							
Mingshui	9	39	14	12	11	21	106
Suihua							
Qiqihar	10	34	18	14	16	32	124
Tailai							
Harbin	14	32	13	11	10	30	110
Nenjiang							
Jixi							
Heihe	8	34	19	10	12	28	111
Huma							
Mudanjiang	11	34	21	12	17	25	120
Shangzhi	7	34	21	10	26	17	115
Suifenhe	9	32	22	11	13	18	105
Tonghe	14	28	24	11	16	22	115
Yilan	13	37	16	12	13	25	116
Baoqing	11	35	17	10	14	25	112
Fujin	10	40	10	12	13	31	116
Hulin	11	32	21	13	12	17	106
Jiamusi	14	37	15	12	15	21	114
Tieli	14	41	17	12	14	24	122

Table S2. Correlation of PET_0 and $SPEI$ with T_{max}, T_{min} and P_r during the rice growth period.

Growth Period	Items	Scenarios	T_{max}(°C)	T_{min}(°C)	P_r(mm/d)
Returning green stage	PET_0	SSP1-2.6	0.882**	0.547**	0.062
		SSP2-4.5	0.899**	0.628**	0.041
		SSP5-8.5	0.944**	0.741**	-0.136
	$SPEI$	SSP1-2.6	-0.176	-0.252*	0.620**
		SSP2-4.5	-0.404**	-0.353**	0.679**
		SSP5-8.5	-0.469**	-0.495**	0.348**
Tillering stage	PET_0	SSP1-2.6	0.904**	0.634**	0.144
		SSP2-4.5	0.905**	0.714**	0.280*
		SSP5-8.5	0.906**	0.756**	0.306**
	$SPEI$	SSP1-2.6	0.072	0.144	0.340**
		SSP2-4.5	-0.090	0.023	0.105
		SSP5-8.5	-0.123	0.031	0.413**
Jointing booting stage	PET_0	SSP1-2.6	0.888**	0.695**	0.157
		SSP2-4.5	0.899**	0.776**	0.192
		SSP5-8.5	0.935**	0.855**	0.235*
	$SPEI$	SSP1-2.6	0.163	0.147	-0.077
		SSP2-4.5	0.423**	0.385**	0.034
		SSP5-8.5	0.223*	0.255*	0.260*
Heading flower stage	PET_0	SSP1-2.6	0.876**	0.750**	0.079
		SSP2-4.5	0.914**	0.809**	0.083
		SSP5-8.5	0.943**	0.872**	0.307**
	$SPEI$	SSP1-2.6	0.260*	0.200	0.280*
		SSP2-4.5	0.031	-0.003	0.006
		SSP5-8.5	0.077	0.085	0.159
Milk stage	PET_0	SSP1-2.6	0.861**	0.667**	-0.023
		SSP2-4.5	0.856**	0.755**	-0.010
		SSP5-8.5	0.891**	0.728**	-0.109
	$SPEI$	SSP1-2.6	0.182	0.177	0.332**
		SSP2-4.5	0.135	0.146	0.074
		SSP5-8.5	0.147	0.158	0.093
Yellow ripening stage	PET_0	SSP1-2.6	0.936**	0.855**	0.521**
		SSP2-4.5	0.937**	0.898**	0.269*
		SSP5-8.5	0.876**	0.839**	0.570**
	$SPEI$	SSP1-2.6	-0.030	-0.040	0.347**
		SSP2-4.5	-0.143	-0.122	0.222
		SSP5-8.5	0.193	0.215	0.399**

Note: * significant correlation at the 0.05 level; ** significant correlation at the 0.01 level.
